# Proteomic characterization of MET-amplified esophageal adenocarcinomas reveals enrichment of alternative splicing- and androgen signaling-related proteins

**DOI:** 10.1007/s00018-025-05635-7

**Published:** 2025-03-13

**Authors:** Bastian Grothey, Su Ir Lyu, Alexander Quaas, Adrian Georg Simon, Jin-On Jung, Wolfgang Schröder, Christiane J. Bruns, Lars M. Schiffmann, Felix C. Popp, Thomas Schmidt, Karl Knipper

**Affiliations:** 1 Faculty of Medicine, Institute of Pathology, University Hospital of Cologne, University of Cologne, Cologne, Germany; 2 Faculty of Medicine, Department of General, Visceral and Cancer Surgery, University Hospital of Cologne, University of Cologne, Cologne, Germany

**Keywords:** Oncology, Esophageal adenocarcinomas, MET signaling pathway, Proteomics, Tumor microenvironment

## Abstract

**Background:**

Esophageal adenocarcinomas (EACs) represent an evolving tumor entity with high mortality rates. MET amplification is a recurrent driver in EACs and is associated with decreased patient survival. However, the response to MET inhibitors is limited. Recent studies have identified several mechanisms that lead to resistance against MET inhibitors in different tumor entities. Nonetheless, a characterization of additional vulnerable targets beyond MET has not been conducted in MET-amplified EACs.

**Methods:**

In this study, we determined the MET amplification status in a cohort of more than 900 EACs using fluorescence in situ hybridization (FISH) and compared the proteomes of MET-amplified (*n* = 20) versus non-amplified tumors (*n* = 39) by mass spectrometry.

**Results:**

We identified a phenotype, present in almost all MET-amplified tumors, which shows an enrichment of alternative RNA splicing, and androgen receptor signaling proteins, as well as decreased patient survival. Additionally, our analyses revealed a negative correlation between MET expression and patient survival in MET-amplified EACs, indicating biological heterogeneity with clinical relevance despite the presence of MET amplification as the predominant oncogenic driver. Furthermore, quantitative immunohistochemical analysis of the inflammatory tumor microenvironment showed that an increased percentage of M2 macrophages is associated with lower overall survival in MET-amplified EACs.

**Conclusions:**

Our results provide valuable insights into possible new therapeutic approaches for MET-amplified EACs for further research.

**Supplementary Information:**

The online version contains supplementary material available at 10.1007/s00018-025-05635-7.

## Introduction

Esophageal adenocarcinoma (EAC) is a significant global public health concern, characterized by its rising incidence and poor prognosis [1]. In advanced stages, the median overall survival is less than one year, with palliative chemotherapy or radiotherapy as recommended treatment options [2, 3]. Recently, treatment options have expanded to include immunomodulatory approaches (programmed death-ligand 1 (PD-L1) inhibitors) and other targeted therapies (human epidermal growth factor receptor 2 (HER2)neu inhibitors). However, these therapies have only slightly improved overall survival [4, 5].

The mesenchymal-epithelial transition (MET) receptor tyrosine kinase regulates cellular processes such as proliferation, migration, and survival through various signaling pathways [6]. In several types of tumors, MET amplifications have been identified as tumor-driving mechanisms that lead to increased activity of the MET signaling pathway [6–9]. In EAC, MET amplifications and increased protein expression correlate with lower overall survival [10, 11]. In recent years, several studies have investigated MET inhibitors in MET-amplified EACs. Although an objective response to therapy was achieved, the median progression-free survival and overall survival were only 3.4 and 7.9 months, respectively [12, 13].

The development of improved therapeutic approaches for MET-amplified tumors requires a detailed understanding of the underlying tumor biology. Previous studies have primarily focused on identifying resistance mechanisms to MET inhibitors. These studies have identified several on-target mutations and off-target events, such as Kirsten rat sarcoma virus (KRAS) gene mutations or HER2neu amplifications, as resistance mechanisms in different tumor entities [14–16]. However, analyses to identify additional vulnerable targets beyond MET, which could serve as the basis for combined therapeutic approaches to mitigate resistance development, have not yet been conducted in MET-amplified EACs.

Proteomic profiling enabled the identification of new subtypes in various cancer entities [17–20]. In a cohort of 124 patients with esophageal squamous cell carcinoma (ESCC), mass spectrometry-based proteomic analyses revealed that the tumor extracellular matrix compared to normal tissue undergoes drastic changes during tumorigenesis. Especially proteins of the cell cycle, DNA repair system, and immune response were enriched in tumors [19]. Additionally, Liu et al. were able to describe two different subtypes with different prognostic implications based on the proteomic analyses in ESCC. Subtype S2 showed compared to S1 and non-tumor tissue upregulated proteins involved in proliferation. Furthermore, a hyperphosphorylation of spliceosomal protein could be detected. These different expressions of proteins involved in cancer cell proliferation and metastasis result in a significantly worse patient survival of patients with subtype S2 [19].

This study aimed to elucidate the differences between MET-amplified and non-amplified esophageal adenocarcinomas on proteome and cellular level. This could lead to further understanding of escape mechanisms of MET-amplified esophageal adenocarcinomas against MET inhibitors as well as identifying additional therapeutic targets beyond MET.

## Materials and methods

### Patients and tumor samples

We included 953 patients with adenocarcinoma of the esophagus who underwent surgery between 2013 and 2019 at the Department of General, Visceral, Cancer, and Transplantation Surgery of the University Hospital Cologne with curative intent. Either a thoracoabdominal esophagectomy with a two-field lymphadenectomy or a transhiatal extended gastrectomy with a D2-lymphadenectomy was performed. The patients of the MET-non-amplified group were chosen randomly from the MET-non-amplified total patient cohort. Here, patients of the MET-amplified and MET-non-amplified group were balanced regarding neoadjuvant therapy and primary surgery. Overall survival was measured from the time of surgery until patient death or loss of follow-up. All included patient data were collected prospectively and analyzed retrospectively. The clinicopathologic assessment of the specimens was conducted following the 7th edition of the Union for International Cancer Control. The study protocol was approved by the local ethical committee (protocol code 20-1393) and conducted in accordance with the Declaration of Helsinki. Written informed consent for inclusion in the databank and tissue bank was obtained from each patient.

To construct the tissue microarray, the tumor tissue was fixed with formalin and embedded in paraffin. Tissue cylinders, 1.2 mm in diameter, were punched from selected areas of the tumors using a semi-automated precision instrument and embedded in paraffin blocks [21].

### Fluorescence-in-situ-hybridization

Fluorescence in situ hybridization (FISH) was performed on the constructed tissue microarray, as previously described [22]. Sections of 4 μm from the paraffin blocks were hybridized overnight with the Zyto-Light SPEC MET/CEN7 Dual Color Probe (ZytoVision, Bremerhaven, Germany). The amplification level was analyzed using a 100x objective and appropriate filter sets (DM5500 fluorescent microscope; Leica Biosystems, Wetzlar, Germany). Twenty tumor cell nuclei were evaluated by counting green (MET) and orange (centromere 7) signals. High amplification was defined, as previously described, as a MET/CEN7 ratio ≥ 2.0, an average MET gene copy number per cell ≥ 6.0, or ≥ 10% of the cells containing ≥ 15 MET signals. Intermediate amplification was defined as ≥ 50% of the cells containing ≥ 5 MET signals, provided none of the criteria for high-level amplification were met [23]. On-slide controls were conducted by screening normal epithelial tissue, fibroblasts, or lymphocytes.

### Immunohistochemistry

We conducted immunohistochemical staining to characterize the tissue microenvironment on whole slide specimens in all included MET-amplified tumors. Staining was performed for the following markers: CD3 for T cells, CD4 for T helper cells, CD8 for cytotoxic T cells, CD20 for B-lymphocytes, CD66b for tumor-associated neutrophils (TANs), CD68 and CD163 for macrophages, mast cell tryptase for mast cells, MUM1 for activated B and T cells as well as plasma cells, and FOXP3 for T regulatory cells, following the manufacturers’ instructions. Additionally, we performed further staining for CD68 and CD163 on the TMAs, encompassing both MET-amplified and non-amplified EACs. Detailed antibody information is provided in Supplementary Table S1. Staining was conducted using the automatic staining system Leica BOND-MAX (Leica Biosystems, Wetzlar, Germany). For each whole slide specimen, twenty fields within the annotated intratumoral or peritumoral region, each measuring 250 × 250 μm, were selected through a randomization process. The peritumoral region was defined as a 300 μm wide radius around the tumor. In these selected fields, cell detection was performed using QuPath Version 0.4.4 [21], allowing for the independent counting of positive and negative cells. For the TMAs, the entire 1.2 mm diameter tissue spot was analyzed. Due to the small size of the TMA cores, no distinction has been made between intratumoral and peritumoral regions. The following metrics were extracted for analysis in our study: number of cell detections, number of negative cells, number of positive cells, percentage positive cells, number of positive cells per mm^2^.

### Proteome analysis with mass spectrometry

Mass spectrometry-based analysis of the proteome was conducted for 59 EACs (20 MET-amplified; 39 MET-non-amplified). A detailed description of the mass spectrometry protocol is provided in the Supplementary materials and methods as described before [24]. To ensure reproducibility, we measured pool samples daily or every 16 patient samples. Before analysis of the samples of our study cohort, we compared the results of the pool samples using a principal component analysis. The results of the measured pool samples formed a cluster, confirming the reproducibility of our mass spectrometry platform. The raw liquid chromatography-mass spectrometry data have been deposited to the ProteomeXchange Consortium via the PRIDE [25] partner repository with the dataset identifier PXD059513. The proteomic dataset has not been published previously.

### Protein-protein interaction network analysis

For the protein-protein interaction network analysis, we constructed a correlation matrix (Pearson’s r) using all available mass spectrometry expression data from the EAC cohort. We then applied several filtering steps. First, we selected only the proteins that were differentially expressed between MET-amplified and non-MET-amplified EACs. Next, we pruned the network by removing interactions with an absolute correlation lower than 0.4. Additionally, for each node (protein), only the three highest absolute correlations were retained, and all other interactions were removed. Finally, for protein cluster identification, we used the Markov Cluster Algorithm (MCL), implemented in the MCL package in R [26].

### Statistical analyses and graphical visualization

The data processing and statistical analyses were conducted using R (version 4.3.2, R Foundation for Statistical Computing, Vienna, Austria). K-means clustering was used to divide the cohort into two groups based on protein expression levels for each protein cluster. Values of the contingency table were analyzed using Fisher’s exact test. For differential expression analysis, we used the limma package [27]. P-values below 0.05 and p-adjusted-values below 0.2 were considered significant. R was also utilized for the graphical visualization of the results. Additionally, Flaticon (https://www.flaticon.com, Freepik Company S.L., Malaga, Spain) was used for further graphical illustrations.

## Results

### Proteomic characterization of MET-amplified esophageal adenocarcinoma reveals distinct survival-associated protein clusters

**Fig. 1:**
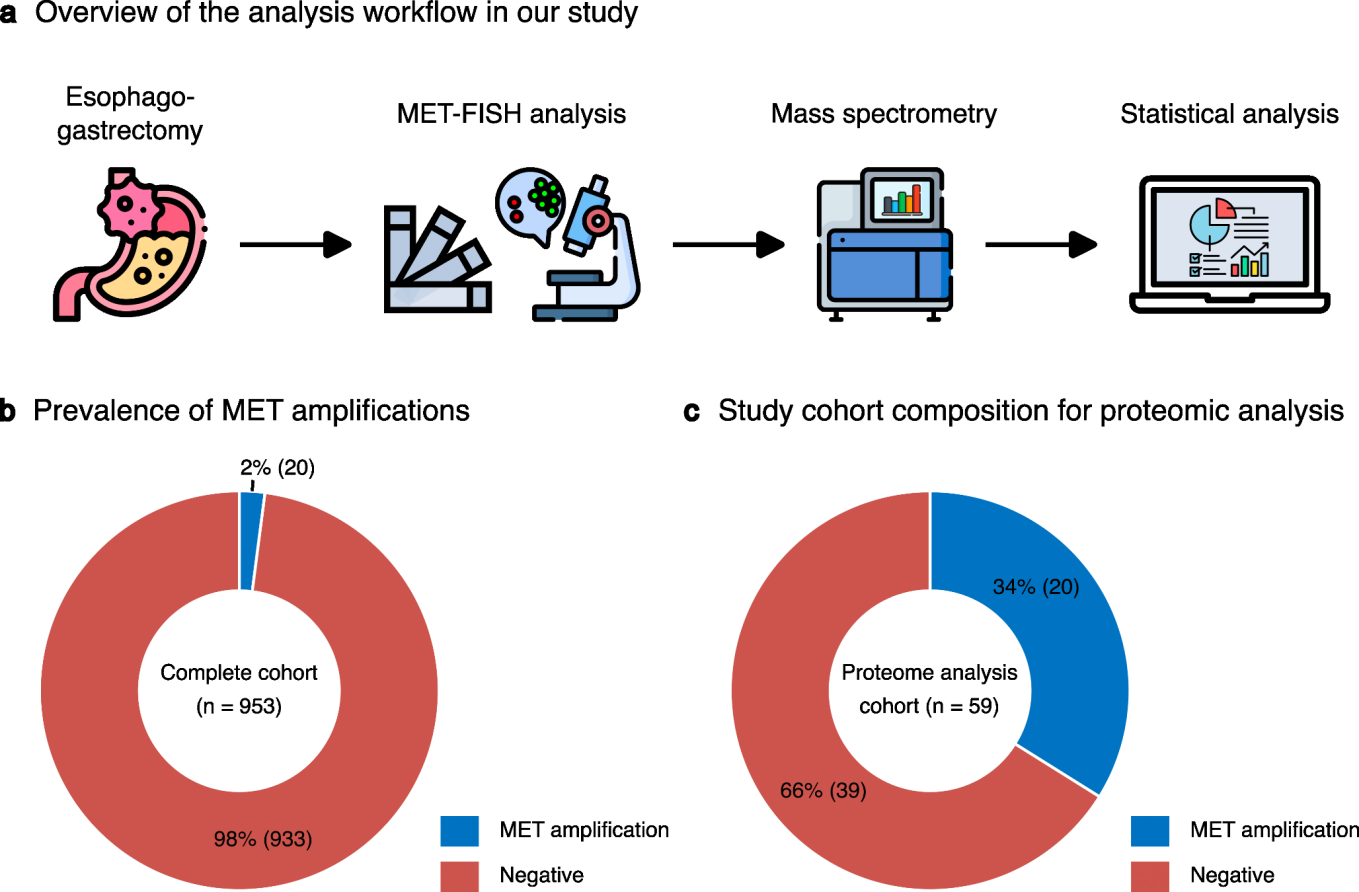
General design and metrics of the project. (**a**) We screened 953 EAC specimens derived from esophagogastrectomy surgeries for MET amplifications using FISH analysis. Twenty EACs exhibited a MET amplification. Together with 39 non-amplified tumors, they were included in a cohort for mass spectrometry-based proteomic analyses followed by quantitative expression characterization. (**b**) Prevalence of MET amplifications in the total cohort consisting of 953 EACs. We detected 2.1% of tumors with a MET amplification. (**c**) Composition of the EAC cohort for proteomic analyses categorized by MET amplification status

We screened 953 patients with EACs who underwent resection of their primary tumors with curative intent for potential MET amplifications using FISH analysis (Fig. 1a). In this cohort, 20 EACs exhibited high MET amplification, corresponding to a prevalence of 2.1% high-level MET-amplified EAC (Fig. 1b). We then employed mass spectrometry-based proteomics to analyze these 20 MET-amplified (MET+) and 39 MET-non-amplified EACs (MET-) to delineate the proteomic landscape associated with MET amplification (Fig. 1c). The general patient characteristics are depicted in Supplementary Table S2. Here, significantly more MET-amplified tumors could be detected in patients over 64 years (*p* = 0.0459).

**Fig. 2:**
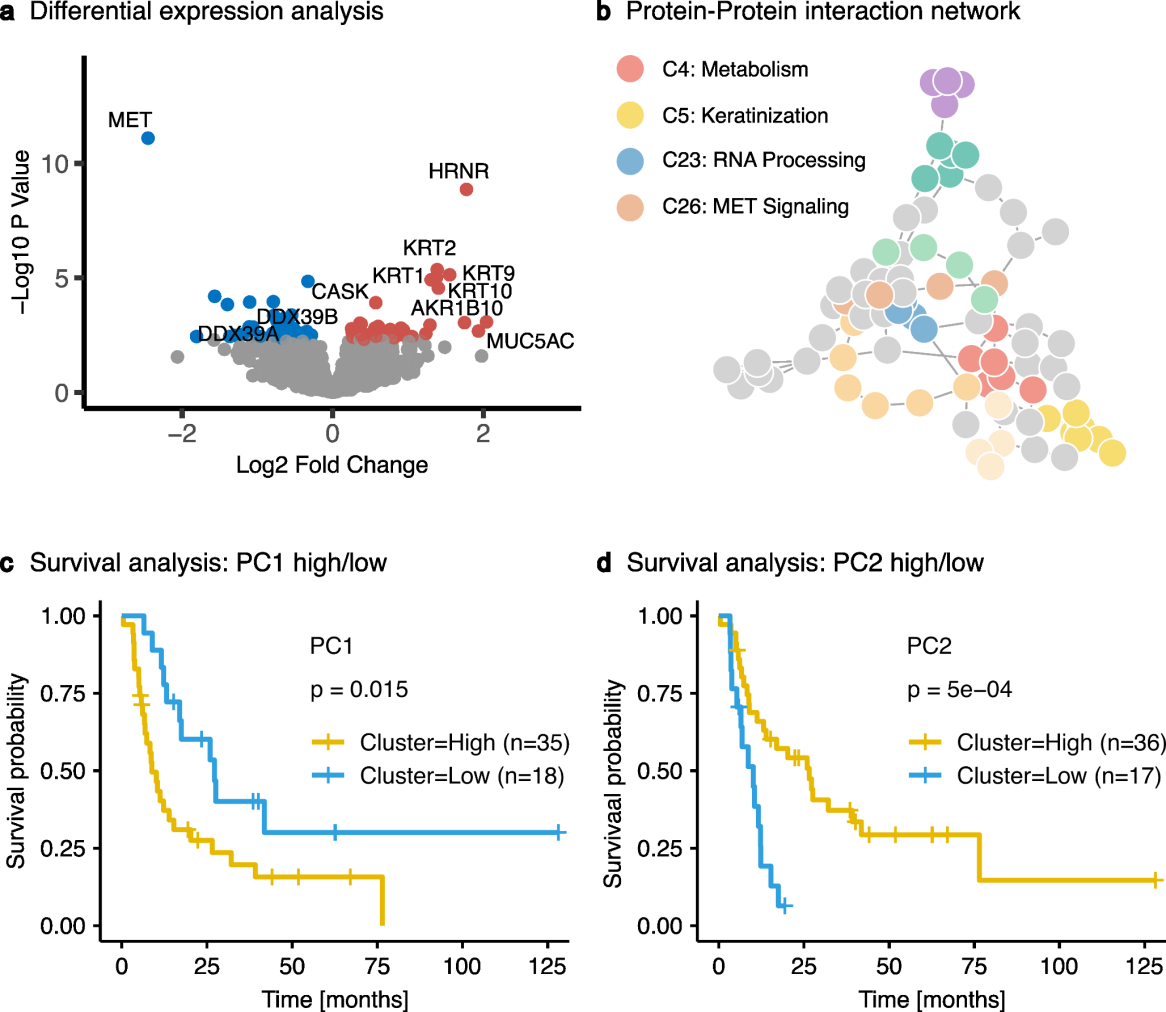
Proteome-based characterization of MET-amplified EACs. (**a**) Differential protein expression analysis for MET-amplified vs. non-amplified EACs (*n* = 59; *p* < 0.05; p-adjusted < 0.2): negative x-axis values indicate higher expression in MET-amplified tumors (blue), while positive values indicate higher expression in non-amplified tumors (red). Grey dots indicate proteins with no significantly different expression. (**b**) Protein-protein correlation network of differentially expressed proteins (MET-amplified vs. non-amplified EACs; *n* = 59; *p* < 0.05; p-adjusted < 0.2) highlighting clusters consisting of more than three proteins. Selected functional associations of the clusters are highlighted on the left. (c + d) Kaplan-Meier survival curves for (**c**) protein cluster 23 (PC1) and (**d**) cluster 4 (PC2), stratified by high and low expression levels determined through k-means clustering (PC1: n_high_ = 35, n_low_ = 18, p_PC1_ = 0.015; PC2: n_high_ = 36, n_low_ = 17, p_PC2_ = 5 × 10^− 4^)

Mass spectrometry-based quantification of protein expression identified 3,277 proteins, enabling an in-depth exploration of proteomic variances. Differential expression analysis between the MET + and MET- groups revealed 81 proteins with significant expression disparities (*p* < 0.05, p-adjust < 0.2) (Fig. 2a; Supplementary Table S3/S4). Confirming the validity of our approach, MET protein exhibited pronounced overexpression in MET + specimens, serving as a conclusive positive control. Notably, a discernible decrease in Hornerin expression was observed in MET + EAC, a protein of the S100 family associated with tumor progression in various entities [28–30]. Additionally, a concomitant reduction in various keratins (KRT1, KRT2, KRT9, KRT10) was recorded.

To understand the biological significance of these differentially expressed proteins, we constructed a correlation-based Protein-Protein Interaction (PPI) Network. We then used the Markov Cluster Algorithm to segregate and delineate functional protein groups indicative of distinct phenotypic signatures. This analysis identified 26 unique protein clusters from the 81 differentially expressed proteins. To ensure heightened phenotypic relevance, we excluded clusters comprising fewer than four proteins from subsequent analyses (Fig. 2b). Overrepresentation analyses (ORA) of these clusters identified pathways associated with various biological processes, including cellular metabolism (cluster 4), keratinization (cluster 5), RNA Processing (cluster 23), and MET signaling (cluster 26), among others (Supplementary Table S5/S6).

Further analysis aimed at elucidating clinically significant phenotypes involved correlating cluster associations with patient survival data. We used the protein expression levels of all proteins within each cluster to perform k-means clustering, dividing the cohort into two groups for each protein cluster. Survival analyses were then conducted to evaluate the prognostic implications. As expected, elevated expression levels of proteins in cluster 26, which includes MET, were significantly correlated with decreased patient survival rates (*p* = 5.9 × 10^-9). Furthermore, the analyses revealed a significant association between decreased survival and increased protein expression in cluster 23 (p_PC1_ = 0.015, Fig. 2c), as well as decreased expression in cluster 4 (p_PC2_ = 5 × 10^− 4^, Fig. 2d), highlighting their potential clinical relevance. Henceforth, these protein clusters are referred to as PC1 (cluster 23) and PC2 (cluster 4). To provide an overview of the proteins in both clusters, Table 1 lists the respective proteins along with a brief description of their functions.

**Table 1:** Overview of the proteins in PC1/PC2 protein cluster with gene names and a brief description of the function

Protein cluster	Gene name	Description
PC1	DDX39A	ATP-dependent RNA helicase DDX39A
PC1	DDX39B	Spliceosome RNA helicase DDX39B
PC1	TBL1XR1	F-box-like/WD repeat-containing protein TBL1XR1
PC1	SPATS2L	SPATS2-like protein
PC2	CASK	Peripheral plasma membrane protein CASK
PC2	AKR1B10	Aldo-keto reductase family 1 member B10
PC2	ALDH1A1	Aldehyde dehydrogenase 1A1
PC2	CA2	Carbonic anhydrase 2
PC2	MUC5AC	Mucin-5AC
PC2	NANS	Sialic acid synthase

### MET amplifications correlate with protein cluster linked to androgen receptor signaling and alternative RNA splicing

**Fig. 3:**
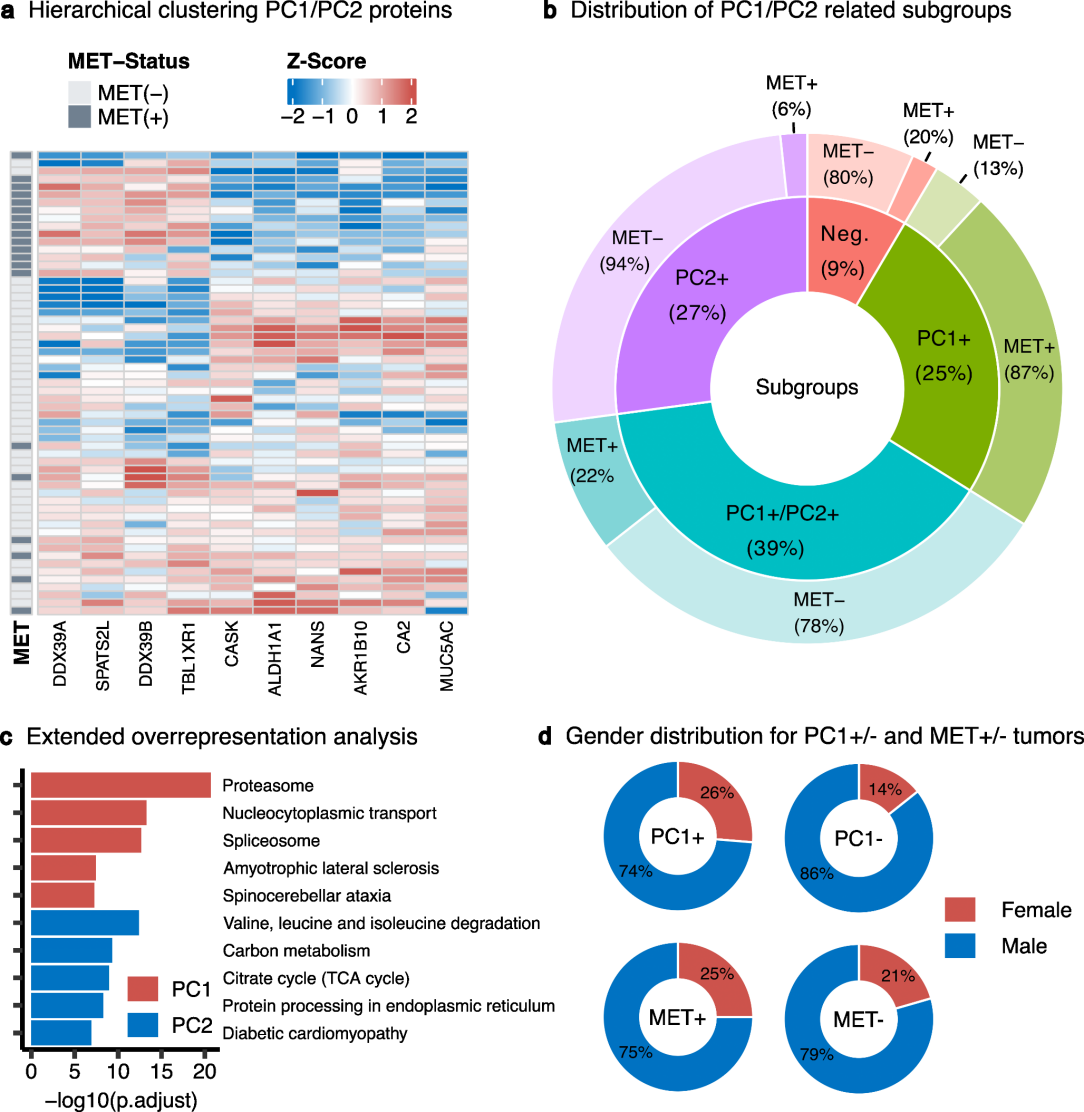
Detailed Characterization of PC1/PC2 Protein Clusters. (**a**) Heatmap depicting PC1/PC2 protein expression levels in MET-amplified and non-amplified EACs (*n* = 59), categorized by MET status. Proteins are grouped into two clusters, PC1 and PC2, as in graph cluster analysis. The heatmap reveals four distinct patterns of protein expression across the tumors: PC1_High_/PC2_High_, PC1_High_/PC2_Low_, PC1_Low_/PC2_High_, and PC1_Low_/PC2_Low_. (**b**) Breakdown of the EAC proteomic cohort (*n* = 59) into four categories based on the expression status (high/low) of PC1 and PC2 proteins (inner circle) and the percentage of MET-amplified/-non-amplified tumors (outer circle). Almost all MET-amplified tumors exhibit high expression of PC1 proteins. Abbreviations: PC1 + = PC1_High_/PC2_Low_, PC2 + = PC1_Low_/PC2_High_, PC1+/PC2 + = PC1_High_/PC2_High_, Neg. = PC1_Low_/PC2_Low_, MET + = MET-amplified, MET- = MET non-amplified. (**c**) Kyoto Encyclopedia of Genes and Genomes (KEGG) Overrepresentation Analysis for PC1 (red) and PC2 (blue) proteins and their correlated protein environment. **(d**) Gender distribution in different subgroups of the analyzed cohort. No significant differences in gender distribution were found for either PC1+/- or MET+/- (Fisher’s exact test: p_PC1_ = 0.47, p_MET_ = 0.78, *n* = 59). Abbreviation: PC1 + = PC1_High_, PC1- = PC1_Low_, MET + = MET-amplified, MET- = MET non-amplified

We conducted an advanced characterization of protein clusters PC1 and PC2. Combined hierarchical clustering of protein expression in both clusters within the EAC cohort segregated the proteins into two groups, aligning with the results of graph cluster analysis (Fig. 3a). Additionally, a binary expression pattern was detected, indicating a further subclassification of the tumors by the two clusters. Utilizing k-means-based categorization of the cohort with individual PC1 and PC2 clusters, as shown in Fig. 2c*/d*, followed by combining the cluster groups, segregated the tumors into four subgroups: PC1_High_/PC2_High_, PC1_High_/PC2_Low_, PC1_Low_/PC2_High_, and PC1_Low_/PC2_Low_ (Fig. 3b). The PC1_High_/PC2_Low_ expression was observed in the majority of MET-amplified tumors (65%, *n* = 13) and only in a few non-amplified tumors, demonstrating a statistically significant enrichment of MET-amplified cases in this subgroup (Fisher’s exact test: PC1_High_/PC2_Low_ vs. the remaining cohort; MET amplified vs. non-amplified, *n* = 59, *p* = 1.5 × 10^− 6^). The remaining MET-amplified tumors predominantly exhibited a high expression pattern for both PC1 and PC2 proteins, with only two instances of low PC1 expression (Fig. 3b). These findings underscore a robust association between the PC1 proteins and MET-amplified EACs.

To further validate and extend our initial observations from the pathway analysis, we performed an overrepresentation analysis on proteins that showed an absolute correlation coefficient greater than 0.4 with the proteins in clusters PC1 and PC2. This analysis aimed to deepen our understanding of the functional profiles and pathways linked to these clusters. The ORA reinforced PC1’s association with RNA processing and splicing activities and revealed an additional link to the proteasome pathway (Fig. 3c; Supplementary Table S7). It also highlighted PC2’s involvement in various metabolic pathways (Fig. 3c; Supplementary Table S8). Furthermore, an in-depth literature review elucidated the connection of PC1 proteins DEAD box RNA helicases 39 A and B (DDX39A/B), and Transducin (β)-like 1 X-linked receptor 1 (TBL1XR1) with androgen receptor (AR) signaling dynamics. Notably, the RNA helicases DDX39A and DDX39B are involved in the generation of the AR splice variant AR-V7 [31], and TBL1XR1 has been identified as a co-activator of AR [32]. This links PC1, and consequently MET amplification, to AR signaling. This connection prompted an investigation into the potential relationship between these molecular subtypes and patient gender. However, Fisher’s Exact Test indicated no significant gender-based differences in the prevalence of PC1 or MET amplification (p_PC1_ = 0.47, p_MET_ = 0.78, Fig. 3d), suggesting that the molecular impact on tumor phenotype does not vary by gender.

### Protein expression, inflammation, and survival link to biological diversity in MET-amplified EACs

**Fig. 4:**
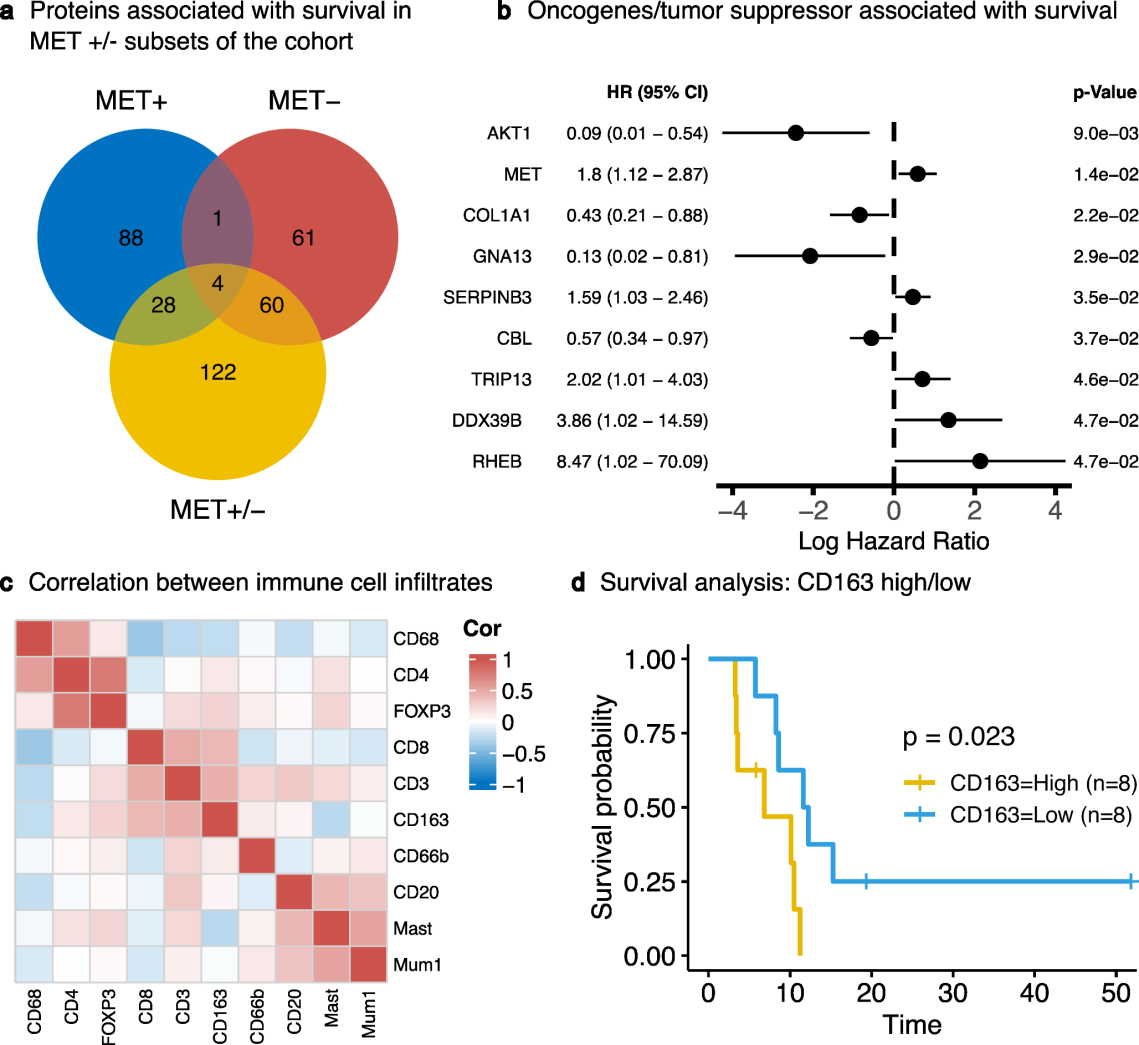
Survival-related parameters in MET-amplified EACs. (**a**) Venn diagram showing the number of proteins associated with survival in Cox regression analysis and their overlaps across different subsets of our study cohort. (significance level: *p* < 0.05; p-adjusted not considered; n_MET+_ = 18; n_MET−_ = 35; n_MET+/−_ = 53) Abbreviations: MET + = only MET-amplified, MET- = only MET-non-amplified, MET+/- = complete dataset. (**b**) Forest plot displaying selected results of Cox regression in MET-amplified EACs (*n* = 18). The plot includes only proteins with a p-value < 0.05 (p-adjusted not considered) which are either annotated as oncogenes/tumor suppressors in the OncoKB database or are part of the protein clusters PC1/PC2 in our analyses. Abbreviations: HR = Hazard ratio, CI = Confidence interval. (**c**) Heatmap showing the correlation among various immune cell markers detected through immunohistochemistry (*n* = 19). The extent of infiltration was measured by the percentage of cells positive for these markers. Three groups of markers with moderate correlation were found: CD4 with FOXP3 and CD68, CD3 with CD8 and CD163, and CD20 with mast cells and MUM1. (**d**) Kaplan-Meier plot of MET-amplified EACs divided into two groups based on high or low percentages of CD163-positive cells (*p* = 0.023, n_High_ = 8, n_Low_ = 8)

Our investigation pivoted to examine survival-related parameters within the cohort of MET-amplified EACs. We aimed to identify proteins whose expression levels are associated with patient survival outcomes. Despite the high number of proteins and the small size of our cohort, raising concerns about multiple testing, we conducted Cox regression analysis on all 3,277 quantified protein expression levels. To provide a comparative overview of the data set, the analyses were carried out in different subsets: MET-amplified (MET+) (Supplementary Table S9), non-amplified (MET-) (Supplementary Table S10), and the complete proteomic data set (MET+/-) (Supplementary Table S11), as depicted in Fig. 4a. As anticipated, none of the proteins showed significant p-adjusted values (p-adjusted < 0.2) due to the extensive number of tests performed. Acknowledging this limitation, we proceeded with further exploratory analyses using unadjusted p-values, applying a significance threshold of *p* < 0.05. This approach uncovered several associations between protein expression and survival across the different subsets. Notably, within the MET-amplified group, 121 proteins were identified as correlated with survival, many of which were uniquely associated with this subset. For a more focused review of clinically relevant proteins, and considering previous findings, we narrowed our analysis to proteins annotated as oncogenes or tumor suppressors in the OncoKB database, or those identified within the PC1 and PC2 protein clusters. This filter reduced the list to nine proteins associated with survival outcomes (Fig. 4b). Among these, AKT1 oncogene expression showed a positive association with increased survival rates (HR = 0.09, 95% CI = 0.01–0.54, *p* = 9 × 10^− 3^). Conversely, even within the MET-amplified cohort, higher MET protein expression was linked to reduced survival (HR = 1.8, 95% CI = 1.12–2.87, *p* = 1.4 × 10^− 2^). A similar observation was made for the helicase DDX39B from the protein cluster PC1 (HR = 3.86, 95% CI = 1.02–14.59, *p* = 4.7 × 10^− 2^). No additional associations of proteins from PC1 and PC2 were identified at the significance level mentioned above.

Finally, our analyses focused on the associations between the tumor microenvironment (TME) and survival in MET-amplified EACs. We evaluated the various markers of inflammation using standard immunohistochemical markers on whole slide specimens of MET-amplified EACs. Correlation analyses among these markers revealed approximately three distinct clusters with moderate inter-marker correlations: CD4/FOXP3/CD68, CD3/CD8/CD163, and CD20/Mast/MUM1 (Fig. 4c).

Univariable Cox regression analysis for each marker identified a significant association between an increased proportion of CD163-positive intratumoral macrophages and poorer survival, underscoring the potential role of macrophages within the TME (Supplementary Figure S1, Supplementary Table S12). Stratifying MET-amplified tumors into high- and low-CD163 groups using a cutpoint finder package [33] corroborated these findings (*p* = 0.023, Fig. 4d). However, no other inflammatory markers demonstrated significant survival associations within our cohort of MET-amplified EACs.

To further explore these findings, we expanded our analyses to CD163-positive macrophages in non-MET-amplified EACs using immunohistochemical staining of our TMAs (see Methods). In addition to CD163 (M2 macrophage marker), we assessed CD68 (M1 macrophage marker) to investigate potential influences of macrophage subpopulations. In total, TMA data from 17 MET-amplified and 787 non-amplified EACs were analyzed. Unlike the whole-slide specimens, no distinction was made between intratumoral and peritumoral regions due to the limited size of the TMA cores. We observed significant associations between CD163/CD68 metrics (positive cell density, percentage of positive cells, total positive cell count) and poorer survival outcomes (Supplementary Table S13).

Taken together, the findings of this section indicate a complex tumor biological heterogeneity with clinical relevance, despite the presence of MET amplification as a predominant oncogenic driver.

## Discussion

Our study provides a comprehensive characterization of the proteome and inflammatory tumor microenvironment (TME) of EACs. First, we screened specimens from 953 patients diagnosed with EAC using FISH to create a cohort of MET-amplified tumors (Fig. 1). Then the proteome of these tumors was analyzed using mass spectrometry and subsequently compared to those of MET non -amplified EACs.

Our results demonstrate that clusters of highly correlated genes with functional similarities are differentially expressed between MET-amplified and non-amplified EACs. We identified four major clusters, each comprising at least four proteins (Fig. 2b). These clusters were assigned functional properties through overrepresentation analysis and literature review: (1) MET signaling, (2) alternative RNA splicing/AR signaling, (3) cellular metabolism, and (4) keratinization. Stratification of the EAC cohort based on the protein expression of these individual clusters revealed a negative association with overall survival for MET and alternative RNA splicing/AR signaling as well as a positive association for cellular metabolism clusters. For the MET signaling cluster, these findings align with previous studies, validating our results and demonstrating that MET activation is detectable in our mass spectrometry data [11].

Notably, combined cluster analyses of alternative RNA splicing/AR signaling (PC1) and the cellular metabolism (PC2) protein clusters revealed four distinct subgroups within the EAC cohort: PC1_High_/PC2_High_, PC1_High_/PC2_Low_, PC1_Low_/PC2_High_, and PC1_Low_/PC2_Low_ (Fig. 3a*/b*). PC1_High_/PC2_Low_ expression was significantly more common in the majority of MET-amplified tumors (65%, *n* = 13) and only in a few non-amplified cases. The remaining MET-amplified tumors predominantly exhibited high expression for both PC1 and PC2 proteins, with only two instances of low PC1 expression, indicating a strong association between PC1 protein expression and MET-amplified EACs. Key proteins of the PC1 cluster include DDX39A/B and TBL1XR1. DDX39A and B are involved in mRNA export from the nucleus into the cytoplasm and splicing [34–36]. Furthermore, DDX39A/B are known for alternative splicing of AR [31]. In prostate cancer, the androgen receptor splice variant-7 (AR-V7) shows ligand-independent activation of the receptor, leading to treatment failure under androgen-suppressing medication in patients with advanced prostate cancer [37]. Downregulation of DDX39A and 39B in AR-V7 expressing cell lines resulted in the suppression of AR-V7 expression [31]. Recent pharmacological studies in prostate cancer have revealed several small molecules that can inhibit AR-V7, thereby decreasing tumor growth in vitro and in vivo [38].

The connection between MET-amplified EAC and AR signaling is further supported by the higher expression of TBL1XR1 in the PC1_High_ subgroup. Besides interacting with various pathways such as Wnt-β-catenin, VEGF, and PI3K/AKT, TBL1XR1 acts as a coactivator of AR [32, 39, 40]. Different observations have been made regarding the significance of TBL1XR1 in malignant tumors. In prostate cancer cell lines, stable ectopic expression of TBL1XR1 was shown to inhibit tumor progression via enhancing AR genes, which exhibit anti-proliferative capabilities [32]. Conversely, gene amplifications of TBL1XR1 were found to be associated with prostate cancer progression [41]. Additionally, TBL1XR1 expression is positively correlated with disease stage in esophageal squamous cell carcinoma and promotes lymphatic metastasis in vitro and in vivo [42]. Despite these associations of cluster proteins with AR signaling, we did not observe a relationship between the phenotype and sex in our data. The significance of AR signaling in EAC has been discussed in recent years [43]. Increased pathway activity correlates with poorer patient survival, and individuals undergoing anti-androgen therapy for prostate carcinoma are less prone to developing esophageal adenocarcinoma [44, 45]. In combination with these literature insights our findings indicate that MET-amplified tumors exhibit alterations in RNA splicing that may affect the functionality of the AR signaling pathway.

The cellular metabolism protein cluster represents a phenotype associated with increased overall survival in our cohort. The simultaneous presence of this phenotype and MET amplifications was observed only in a limited number of cases and almost exclusively alongside high expression of PC1 proteins. Overall, this phenotype appears to be complex and seems to represent a metabolic condition that plays a secondary role in MET-amplified tumors. The cluster includes proteins linked to the citric acid cycle, amino acid degradation, and protein processing. In gastric cancer, the upregulation of citric acid cycle proteins has been described in a subgroup of patients [46]. In these patients, the upregulation of related genes correlated with improved overall survival. Furthermore, these patients showed a better response to immune checkpoint inhibitors [46]. Additionally, various research efforts have targeted the citric acid cycle as a potential treatment option [47]. Our results highlight the relevance of the metabolome as a potential target for new therapeutic approaches in EACs. Further research with more focused analyses is necessary to better understand tumor vulnerabilities in this area.

The remaining keratinization cluster, which is not associated with overall survival, includes several keratins (KRT1/2/9/10) and Hornerin, a member of the S100 protein family, which were significantly downregulated in MET-amplified EACs. Hornerin, part of the S100 protein family, has been linked to tumor progression in various entities [28–30]. Interestingly, Hornerin is highly expressed on endothelial cells in pancreatic adenocarcinoma in a VEGF-independent manner, and in vivo knockdown resulted in smaller vessels and reduced tumor growth [30]. However, a comprehensive functional characterization, especially of the role of the protein in malignant tumors, has not yet been conducted, and additional research is required.

In the second part of our study, we focused on risk factors within the group of MET-amplified tumors, analyzing both proteomic data and the inflammatory TME using immunohistochemical methods. Our proteomic data indicated that even within MET-amplified tumors, higher MET expression correlates with lower overall survival. Similarly, DDX39B, part of the PC1 cluster, showed the same trend. Conversely, higher expression of the oncogene AKT was associated with an increased overall survival. However, it’s important to acknowledge that these findings are exploratory. Testing 3,277 proteins in a relatively small cohort raises concerns about multiple testing, and the adjusted p-values did not reach statistical significance. Analyses of immune infiltrating cells in the TME of MET-amplified EACs revealed that a higher percentage of intratumoral M2 macrophages (CD163) correlates with lower overall survival. The correlation of M2 macrophages with poor patient survival has been previously described in esophageal adenocarcinoma [48]. Our study confirmed this finding in MET-amplified tumors, providing a more detailed insight into the prognostically significant immune infiltrating cells in the MET-amplified tumor microenvironment. Immunohistochemical stainings of the macrophage markers CD68 and CD163 on our TMAs revealed that a higher amount of infiltrating CD68- or CD163-positive cells is associated with worse patient survival. These results are in line with previous studies [48, 49]. The composition of the inflammatory infiltrate plays a crucial role in cancer progression [50]. Notably, hepatocyte growth factor (HGF), the ligand for the MET receptor, modulates cytokine expression patterns in macrophages. Specifically, bone marrow-derived macrophages secrete fewer pro-inflammatory cytokines and more anti-inflammatory cytokines in the presence of HGF [51]. Furthermore, activation of MET shifts macrophages from the pro-inflammatory M1 phenotype to an M2-like phenotype [52]. M2 macrophages are known to act in a protumorigenic manner, suggesting that MET activation may contribute to tumor progression through a shift of macrophage polarization [53]. Nevertheless, to develop therapeutic concepts, further mechanistic research is needed to investigate the underlying biological processes. In summary, our findings from the second part of our study highlight the complex tumor heterogeneity with potential clinical implications despite the presence of MET amplification as a predominant oncogenic driver.

Our study is not without limitations. Firstly, its descriptive design may limit the clinical significance of the findings. Further mechanistic experiments are essential to substantiate the hypotheses proposed here. Secondly, our investigation focused solely on the proteome level using mass spectrometry and partially examined the inflammatory TME through immunohistochemistry. Other cell types, such as fibroblasts, might also play a role in the progression of MET-amplified tumors and should be investigated in future studies. Thirdly, in addition to protein expression, other factors like mutations, copy number changes, and phosphorylation status, which are crucial for the signaling networks in MET-amplified tumors [54], were not analyzed in this study. Lastly, our survival analyses are constrained by the retrospective study design and the relatively low number of patients. Prospective studies are needed to confirm our hypotheses and findings.

## Conclusions

Our study provides a detailed characterization of the proteome and the inflammatory TME of MET-amplified EACs. We identify a distinct phenotype significantly overrepresented in MET-amplified EACs, characterized by high expression of proteins associated with alternative RNA splicing and AR signaling. Additionally, by analyzing the tumor microenvironment of MET-amplified EACs, we demonstrate that an increased percentage of M2 macrophages is associated with lower overall survival. Although further studies are necessary to elucidate the underlying mechanisms, our findings offer valuable insights for future research on new possible therapeutic approaches for MET-amplified EACs.

## Electronic supplementary material

Below is the link to the electronic supplementary material.

Supplementary Methods

Supplementary Figure S1. Exemplary pictures of immunohistochemical staining with (a) low and (b) high CD163 expression. Sidebar: 50 µm.

Supplementary Table S2. General patient characteristics of the total cohort and MET-amplified or non-amplified subcohorts. Differences between groups were evaluated for statistical significance using Fisher’s exact test. CROSS: Chemoradiotherapy for Oesophageal Cancer Followed by Surgery Study; FLOT: Fluorouracil, Leucovorin, Oxaliplatin, Docetaxel; pT: Histopathological T-stage; pN: Histopathological N-stage; L: Lymphovascular invasion; V: Vascular invasion; Pn: Perineural invasion. Bold print marks p-value below 0.05.

Supplementary Table S1. Detailed information of all used antibodies for immunohistochemical stainings. A: appendix vermiformis, T: tonsilla palatina.

Supplementary Table S3. Protein expression levels in MET-amplified and non-amplified esophageal adenocarcinoma samples. The table contains processed protein expression data from mass spectrometry-based proteomic analysis. It represents a subset of a raw mass spectrometry dataset we deposited on PRIDE database with the samples we used in this study. Each row contains a unique protein identified by its UniProt identifier (UniProtID). Column headers indicate the amplification status of each sample (amplified or non_amplified) followed by a number. Note that these do not represent the sample IDs we used in the raw data deposited in PRIDE database.

Supplementary Table S4. Detailed results of differential expression analysis (MET-amplified vs non-amplified EACs; n = 59) using R package limma. Negative logFC values indicate higher expression in MET-amplified tumors, while positive values indicate higher expression in non-amplified tumors. No filters were applied.

Supplementary Table S5. KEGG overrepresentation analyses of clusters derived from protein-protein interaction network analysis. Each protein cluster was analyzed separately. Cluster_ID = Protein cluster ID; ID = KEGG pathway ID.

Supplementary Table S6. Reactome overrepresentation analyses of clusters derived from protein-protein interaction network analysis. Each protein cluster was analyzed separately. Cluster_ID = Protein cluster ID; ID = REACTOME pathway ID.

Supplementary Table S7. KEGG overrepresentation analysis of combined PC1 cluster proteins and correlated proteins. Proteins that showed an absolute correlation coefficient greater than 0.4 with the proteins in clusters PC1 were included. A highly statistically significant association is observed with the Proteasome pathway. ID = KEGG pathway ID.

Supplementary Table S8.KEGG overrepresentation analysis of combined PC2 cluster proteins and correlated proteins. Proteins that showed an absolute correlation coefficient greater than 0.4 with the proteins in clusters PC2 were included. Several associations to pathways linked to cellular metabolism can be observed. ID = KEGG pathway ID.

Supplementary Table S9. Detailed results of univariable Cox regression on MET-amplified EACs examine the relationship between survival and protein expression for all proteins derived from mass spectrometry. HR = Hazard ration; LowerCI = Lower confidence interval; UpperCI = Upper confidence interval.

Supplementary Table S10. Detailed results of univariable Cox regression on EACs without MET amplification examine the relationship between survival and protein expression for all proteins derived from mass spectrometry. HR = Hazard ration; LowerCI = Lower confidence interval; UpperCI = Upper confidence interval.

Supplementary Table S11. Detailed results of univariable Cox regression on the proteomic data set including amplified and non-amplified EACs examine the relationship between survival and protein expression for all proteins derived from mass spectrometry. HR = Hazard ration; LowerCI = Lower confidence interval; UpperCI = Upper confidence interval.

Supplementary Table S12. Detailed results of univariable Cox regression on MET-amplified EACs examine the relationship between survival and the inflammatory tumor microenvironment, quantified by immunohistochemistry analyses on whole slide specimens (n=17). HR = Hazard ration; LowerCI = Lower confidence interval; UpperCI = Upper confidence interval.

Supplementary Table S13. Detailed results of univariable Cox regression on MET-amplified EACs examine the relationship between survival and CD68/CD163 positive cells quantified by immunohistochemistry analyses on our TMAs, including 17 MET-amplified and 787 non-amplified EACs (n = 804). HR = Hazard ration; LowerCI = Lower confidence interval; UpperCI = Upper confidence interval.

## Data Availability

The underlying code of the study is available upon request.

## Abbreviations

AR: Androgen receptor
AR-V7: Androgen receptor splice variant-7
CD: Cluster of differentiation
CI: Confidence interval
CROSS: Chemoradiotherapy for Oesophageal Cancer Followed by Surgery Study
DDX39: DEAD box RNA helicases 39
DNA: Deoxyribonucleic acid
EAC: Esophageal adenocarcinoma
ESCC: Esophageal squamous cell carcinoma
et al.: et alia (and others)
FISH: Fluorescence in situ hybridization
FLOT: Fluorouracil, Leucovorin, Oxaliplatin, Docetaxel
FOXP3: Forkhead box P3
HER2: Human epidermal growth factor receptor 2
HGF: Hepatocyte growth factor
HR: Hazard Ratio
KEGG: Kyoto Encyclopedia of Genes and Genomes
KRAS: Kirsten rat sarcoma virus
KRT: Kreatin
Mast: Mast cell tryptase
MCL: Markov Cluster Algorithm
MET: Mesenchymal-epithelial transition
MET+: MET-amplified
MET-: MET-non-amplified
µm: Micrometer
mm: Millimeter
mRNA: Messenger ribonucleic acid
MUM1: Interferon regulatory factor 4
ORA: Overrepresentation analyses
PC: Protein cluster
PD-L1: Programmed death-ligand 1
PPI: Protein-Protein Interaction
RNA: Ribonucleic acid
TBL1XLR1: Transducin (β)-like 1 X-linked receptor 1
TMA: Tissue microarray
TME: Tumor microenvironment
TNA: Tumor-associated neutrophils
VEGF: Vascular endothelial growth factor
vs: Versus

## Data availability

The datasets generated and analyzed during the current study are available from the corresponding author upon reasonable request.
