# Supplementary material for: Proteomic characterization of MET-amplified esophageal adenocarcinomas reveals enrichment of alternative splicing- and androgen signaling-related proteins
Source: Cell Mol Life Sci. 2025 Mar 13;82(1):112. doi: 10.1007/s00018-025-05635-7 (PMC11904063; doi:10.1007/s00018-025-05635-7)
Supplement: Supplementary file 1 — Supplementary Methods [file 18_2025_5635_MOESM1_ESM.docx]

**Supplementary materials and methods**

*FFPE-tissue lysis and digestion*

Samples were arranged in a randomized order that was followed for the rest of the experiment including protein extraction, digestion, and LC–MS acquisition. Two hundred microliters of cooled phosphate-buffered saline containing 1 x Halt Protease Inhibitor Cocktail (Thermo Fisher Scientific, Waltham, MA, USA) was added to a mixture of 1.4 mm and 2.8 mm ceramic beads in 2 ml lysis tubes (Bertin Technologies SAS, Montigny-le-Bretonneux, France). Two FFPE tumor sections per sample were deparaffinized and added to the vials, and homogenized with two 20 s cycles at 5,800 rpm in a precooled Precellys ball mill (Bertin Technologies). After homogenization, the tubes were centrifuged for 5 min at 20,000 x*g*, 4 °C. Then 190 μl of supernatant was transferred to a fresh 0.5 ml tube. After the addition of 50 µl of 20% SDS and 12 µl of dithiothreitol (DTT), the samples were heated to 95 °C for 60 min and sonicated using a Bioruptor (Diagenode, Denville, NJ, USA) with 10 x 30 s pulses and a 30 s delay between pulses. The heating and sonication sequence was repeated one more time, then 24.2 µl of 400 mm chloroacetamide in water was added and the samples were incubated for 30 min at room temperature in the dark. Samples of protein lysates (50 µl) were used for protein digestion according to the single-pot solid-phase-enhanced sample preparation protocol ^1^. After the final peptide clean up, the beads were reconstituted in 9 µl of 5% DMSO. After 10 min, the magnetic beads were trapped again, and supernatants were transferred to fresh tubes and acidified by the addition of 1 µl of 10% formic acid.

*Liquid chromatography–mass spectrometry* (*LC–MS*)

Peptides were analyzed in a Q Exactive Exploris 480 mass spectrometer equipped with a FAIMS Pro differential ion mobility device that was coupled to an EASY-nLC 1200 nano HPLC system (both Thermo Fisher Scientific). A pulled tip 75 µm x 300 mm column packed in house with Poroshell C18 2.7 μm (Agilent Technologies, Santa Clara, CA, USA) was placed in a column oven (Sonation, Biberach, Germany) and operated at 50 °C and a flow rate of 300 nl/min. After pre-equilibration in buffer A (0.1% FA), 3 µl of the acidified protein digests was injected and peptides were separated using a gradient of 2–6% buffer B (0.1% FA in 80% ACN) for 1 min, followed by 6–30% buffer B for 72 min, 30–55% for 7 min, 55–95% for 2 min, and 8 min final column regeneration at 95%. The FAIMS Pro was operated at −50 V compensation voltage and electrode temperatures of 99.5 °C for the inner and 85 °C for the outer electrode. Identical HPLC settings were used for library generation and sample runs.

Spectrum library generation by gas phase fractionation: Aliquots from each sample were pooled and the pool was used for spectrum library generation by narrow window DIA of six 100 *m*/*z* gas phase fractions (GPFs) covering the range from 400 *m*/*z* to 1000 *m*/*z* ^2^. The Orbitrap was operated in DIA mode. MS1 scans of the respective 100 *m/z* gas phase fraction were acquired at 30k resolution. Maximum injection time was set to 50 ms and the AGC target to 100%. MS2 scans of the corresponding 100 *m/z* region were acquired in 24 x 4 *m/z* staggered windows, resulting in 48 nominal 2 *m/z* windows after demultiplexing. MS2 settings were 15k resolution, 55 ms maximum injection time, and an AGC target of 1,000%. All scans were stored as a centroid.

Data independent acquisition of samples: MS1 scans were acquired from 390 *m/z* to 1010 *m/z* at 15k resolution. Maximum injection time was set to 25 ms and the AGC target to 100%. MS2 scans ranged from 300 *m/z* to 1500 *m/z* and were acquired at 15k resolution with a maximum injection time of 22 ms and an AGC target of 1,000%. DIA scans covering the precursor range from 400 to1000 *m/z* were acquired in 75 x 8 *m/z* staggered windows, resulting in 150 nominal 6 *m/z* windows after demultiplexing. All scans were stored as a centroid. To minimize batch effects, the acquisition was performed in a randomized order.

*Primary processing of raw files*

Thermo raw files were demultiplexed and transformed to mzML files using the msConvert module in ProteoWizard ^3^. For spectral library generation, a human canonical Swiss-Prot fasta file was converted to a Prosit upload file using the convert tool in encyclopedia 0.9.0 with default settings ^2^. Trypsin, up to one missed cleavage, range 396–1004 *m/z*, charge states 2+ and 3+, default charge state 3, and NCE 33. The csv file was uploaded to the Prosit webserver and converted to a spectrum library in generic text format ^4^. The resulting library (20,374 proteins, 20,083 genes, and 1,626,266 precursors) was searched in DIA-NN 1.7.12 with the six GPF runs to generate the project specific library (9,634 proteins, 9,539 genes, and 57,711 precursors) ^5^. The applied settings were as follows: Output will be filtered at 0.01 FDR; N-terminal methionine excision enabled; Maximum number of missed cleavages set to 1; Min peptide length set to 7; Max peptide length set to 30; Min precursor *m/z* set to 400; Max precursor *m/z* set to 1,000; Cysteine carbamidomethylation enabled as a fixed modification. Sample files were searched using DIA-NN 1.7.12 with the project library. In addition to the settings used for library generation, grouping on protein names from fasta file and retention time-dependent normalization were used. Sample runs with precursor ID numbers outside the quartiles plus/minus the inter-quartile range were regarded as outliers and removed (7 out of 146). For quantitative analysis, precursor identifications were first filtered for at least four fragment ions, *q* values < 0.01, and library *q* values < 0.005. Normalized intensities of precursor ions corresponding to proteotypic peptides were selected for the calculation of protein intensities using the MaxLFQ algorithm implemented in the DIA-NN R-package. To analyze for potential processing or acquisition-related batch effects, principal component analysis of protein intensities was plotted and colored corresponding to technical features, such as digest batch, acquisition order, instrument calibration dates, FAIMS cleaning dates, and changes of chromatography columns.

Proteins detected in at least 70% of samples in any SGC tumor subtype were included in further analyses. Imputation was carried out on log2 normalized intensities using the ‘Ensembl’ output of the DreamAI R (R Foundation for Statistical Computing, Vienna, Austria) package from imputations with methods ‘KNN’, ‘MissForest’, ‘Birnn’, ‘SpectroFM’, and ‘RegImpute’ (Preprint ^6^). Still a preprint, it has been used and featured in high-impact publications and has also been successfully applied using unlabelled LC–MS ^7,8^.

**References**

1 Hughes, C. S. *et al.* Single-pot, solid-phase-enhanced sample preparation for proteomics experiments. *Nat Protoc* **14**, 68-85, doi:10.1038/s41596-018-0082-x (2019).

2 Searle, B. C. *et al.* Chromatogram libraries improve peptide detection and quantification by data independent acquisition mass spectrometry. *Nat Commun* **9**, 5128, doi:10.1038/s41467-018-07454-w (2018).

3 Chambers, M. C. *et al.* A cross-platform toolkit for mass spectrometry and proteomics. *Nat Biotechnol* **30**, 918-920, doi:10.1038/nbt.2377 (2012).

4 Gessulat, S. *et al.* Prosit: proteome-wide prediction of peptide tandem mass spectra by deep learning. *Nat Methods* **16**, 509-518, doi:10.1038/s41592-019-0426-7 (2019).

5 Demichev, V., Messner, C. B., Vernardis, S. I., Lilley, K. S. & Ralser, M. DIA-NN: neural networks and interference correction enable deep proteome coverage in high throughput. *Nat Methods* **17**, 41-44, doi:10.1038/s41592-019-0638-x (2020).

6 Ma W, K. S., Chowdhury S. DreamAI: algorithm for the imputation of proteomics data. *bioRxiv*, doi:2020.07.21.214205 (2020).

7 Mani, D. R. *et al.* Cancer proteogenomics: current impact and future prospects. *Nat Rev Cancer* **22**, 298-313, doi:10.1038/s41568-022-00446-5 (2022).

8 Ebner, J. N., Wyss, M. K., Ritz, D. & von Fumetti, S. Effects of thermal acclimation on the proteome of the planarian Crenobia alpina from an alpine freshwater spring. *J Exp Biol* **225**, doi:10.1242/jeb.244218 (2022).
