# Supplementary figures and images for: Proteomic characterization of MET-amplified esophageal adenocarcinomas reveals enrichment of alternative splicing- and androgen signaling-related proteins
Source: Cell Mol Life Sci. 2025 Mar 13;82(1):112. doi: 10.1007/s00018-025-05635-7 (PMC11904063; doi:10.1007/s00018-025-05635-7)

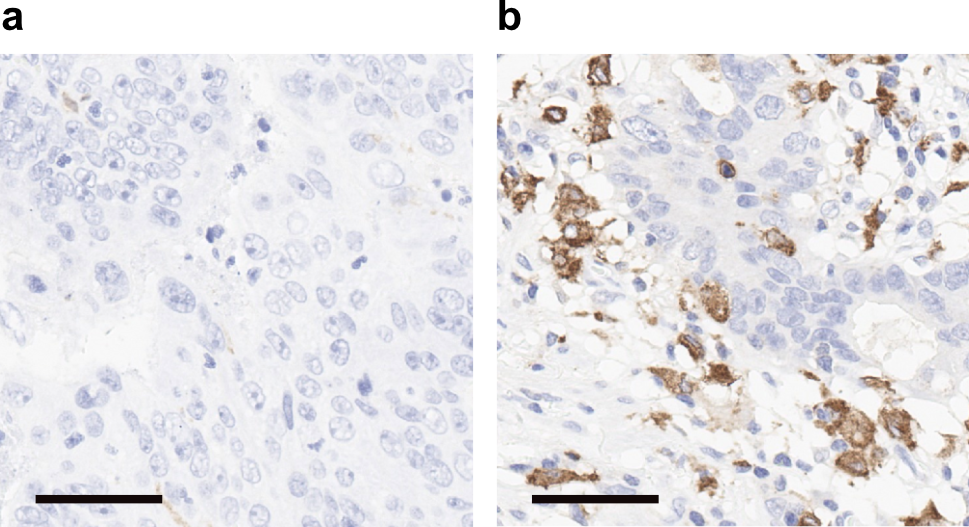

Supplement: Supplementary file 2 — Supplementary Figure S1. Exemplary pictures of immunohistochemical staining with (a) low and (b) high CD163 expression. Sidebar: 50 µm. [file 18_2025_5635_MOESM2_ESM.tiff]
